# Global Hotspots and Trends in Microbial Plastic Biodegradation for Plastic Waste Management: A Mini-Review and Bibliometric Analysis

**DOI:** 10.3390/microorganisms14081695

**Published:** 2026-08-02

**Authors:** Haibo Wang, Zhikang Guo, Yunan Liu, Hao Shen, Fang Chen, Mu Peng

**Affiliations:** 1 Hubei Key Laboratory of Biological Resources Protection and Utilization, Hubei Minzu University, Enshi 445000, China; wanghaibo0519@126.com (H.W.); 15072395917@163.com (Z.G.); 2023033@hbmzu.edu.cn (H.S.); 2 College of Biological and Food Engineering, Hubei Minzu University, Enshi 445000, China; 2024322040039@stu.scu.edu.cn; 3 School of Cybersecurity Governance, Cyberspace Security University of China, Wuhan 430040, China; liuyunan@csuc.edu.cn; 4 College of Life Science, Sichuan University, Chengdu 610065, China

**Keywords:** microbial plastic biodegradation, plastic-degrading enzymes, microplastics, bibliometric analysis, Web of Science Core Collection

## Abstract

The accumulation and persistence of plastic waste have made microbial plastic biodegradation an important topic in pollution control, environmental remediation, and sustainable materials management. This study combines a mini-review with bibliometric analysis to link mechanistic understanding with global research trends in microbial plastic biodegradation from 2000 to 2025. The mini-review summarizes polymer weathering and fragmentation, microbial colonization and biofilm formation, extracellular depolymerization or oxidative chain cleavage, uptake and intracellular catabolism of plastic-derived intermediates, physiological regulation, ecological interactions, and potential applications in bioremediation and upcycling. Bibliographic records were retrieved from the Web of Science Core Collection and analyzed using bibliometrix, VOSviewer, CiteSpace, and SCImago Graphica. A total of 2959 publications were identified. Publication output increased markedly, especially after 2018, reaching 617 publications in 2025; cumulative citations reached 31,373. China, India, and the United States were the leading contributors. Journal and keyword analyses showed strong links among environmental science, polymer science, microbiology, biotechnology, and engineering. Highly cited publications mainly focused on plastic biodegradability, biodegradable polymers, engineered PET depolymerization, polyethylene degradation, and microbial or enzymatic degradation mechanisms. Keyword evolution revealed a shift from material-oriented topics, including polymer blends, poly(vinyl alcohol), polyesters, morphology, mechanical properties, composites, and polyhydroxyalkanoates, toward degrading enzymes, cutinase-like enzymes, plastic-degrading strains, microbial colonization, fungi, and marine environmental degradation. Overall, microbial plastic biodegradation has evolved from material-centered biodegradability evaluation toward a mechanism-oriented and environment-oriented interdisciplinary field.

## 1. Introduction

**Figure 1:**
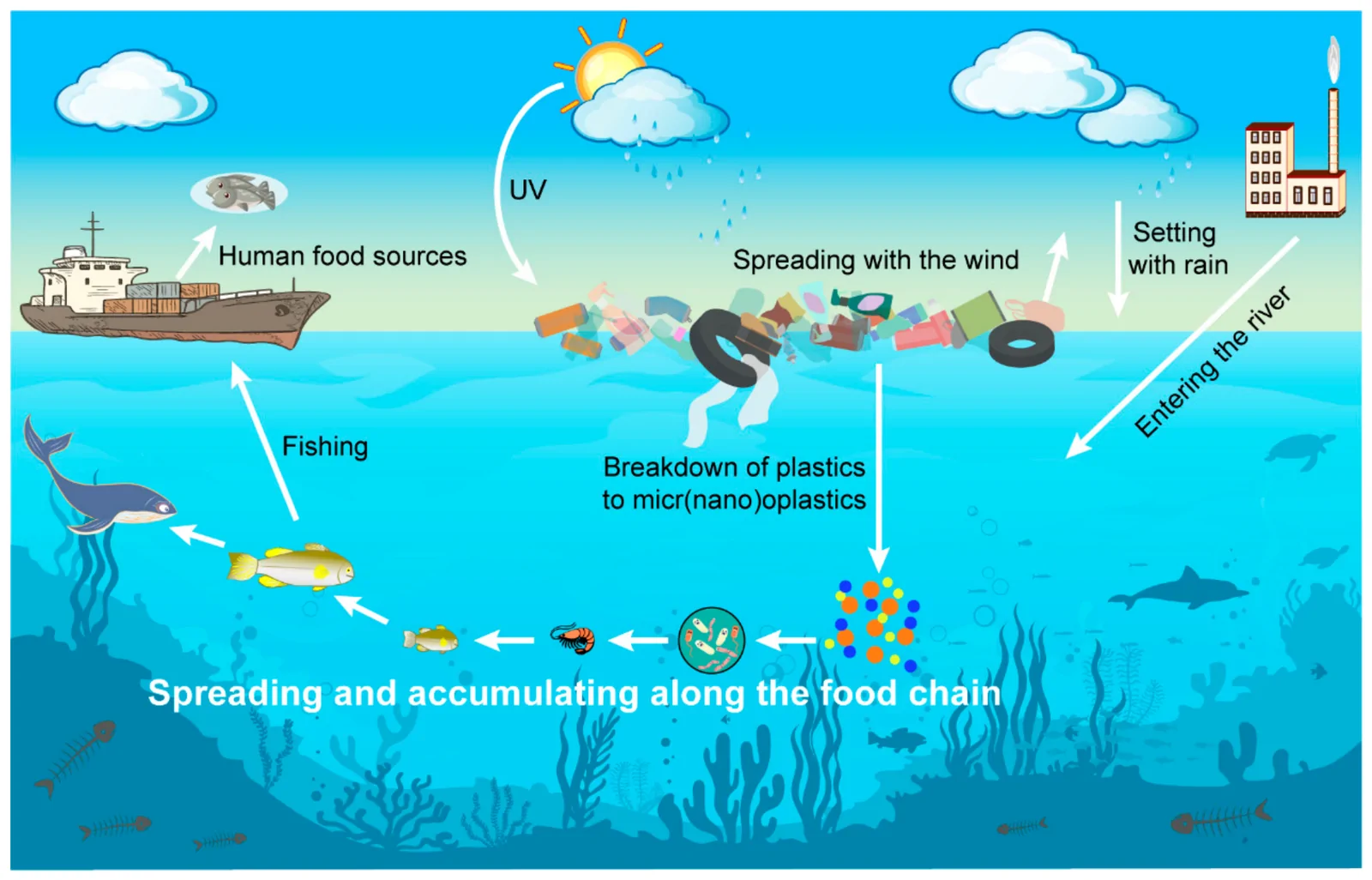
Plastic fragmentation, micro- and nanoplastic transport, biological accumulation, and trophic transfer in aquatic food webs.

The accumulation and long-term environmental persistence of plastic waste have become major concerns in environmental pollution control, ecosystem protection, and sustainable materials management [1,2]. Once released into the environment, plastics can enter terrestrial, freshwater, and marine ecosystems through runoff, atmospheric deposition, river transport, wastewater discharge, and improper waste management. Under physical abrasion, photodegradation, thermo-oxidation, and chemical weathering, large plastic debris can gradually age and fragment into microplastics and nanoplastics [3]. These particles can be transported across environmental compartments by wind, rainfall, rivers, and ocean currents [4,5], and may enter aquatic food webs through ingestion by plankton, benthic organisms, mollusks, crustaceans, fish, seabirds, and other organisms [6,7]. Therefore, plastic pollution should be regarded not only as a problem of material persistence, but also as a complex environmental issue involving fragmentation, environmental transport, biological uptake and accumulation, trophic transfer, and potential human exposure [8,9] (Figure 1).

Microbial plastic biodegradation provides an important biological route for understanding and potentially mitigating plastic pollution [10]. Plastic surfaces can serve as artificial habitats for bacteria, fungi, microalgae, and other microorganisms, while weathering-induced surface roughness and oxidation can promote microbial attachment and biofilm formation [11,12,13]. Within plastic-associated biofilms, microorganisms may mediate extracellular depolymerization through extracellular or surface-associated enzymes [14,15]. For ester-containing plastics such as polyethylene terephthalate (PET), poly(lactic acid) (PLA), poly(ε-caprolactone) (PCL), and polyester-type polyurethanes, cutinases, esterases, and lipases can cleave ester bonds, releasing oligomers and monomers [16,17]. By contrast, plastics with carbon–carbon backbones, such as polyethylene (PE), polypropylene (PP), and polystyrene (PS), are generally more resistant to biodegradation. Abiotic oxidation may introduce oxygen-containing functional groups and generate low-molecular-weight oxidized products that can be further transformed by microorganisms; however, complete microbial mineralization remains limited and highly dependent on polymer type, environmental conditions, and microbial community composition [18,19]. These plastic-derived compounds may then be taken up by microbial cells and channeled into intracellular catabolism and central carbon metabolism, supporting energy production, biomass formation, mineralization, or conversion into value-added products [20].

With the expansion of this field, microbial plastic biodegradation research has shifted from determining whether plastics can be degraded to investigating plastic-degrading microorganisms and enzymes, extracellular depolymerization, intracellular metabolism, ecological risks, and application-oriented bioremediation and upcycling strategies [21]. Because this topic spans polymer science, microbiology, environmental science, materials science, and resource management, bibliometric analysis can systematically reveal its developmental trajectory, knowledge structure, collaboration patterns, and emerging hotspots [22].

In this study, we combine a concise mechanistic review with a global bibliometric analysis of microbial plastic biodegradation research from 2000 to 2025. The bibliometrix package 4.5.0, VOSviewer (version 1.6.20), CiteSpace (version 7.0.R0), Microsoft Excel, and SCImago Graphica (version 1.0.43) were used to analyze publication trends, disciplinary distributions, collaboration networks, core journals, highly cited references, keyword co-occurrence structures, and emerging frontiers. By integrating mechanistic synthesis with bibliometric mapping, this study aims to clarify the biological significance, global research evolution, and future directions of microbial plastic biodegradation, and to provide a reference for research on efficient plastic-degrading microorganisms and enzymes, degradation mechanisms under environmentally realistic conditions, ecological safety, plastic bioremediation, and circular resource utilization.

## 2. Mini-Review: Mechanisms of Microbial Plastic Biodegradation

**Figure 2:**
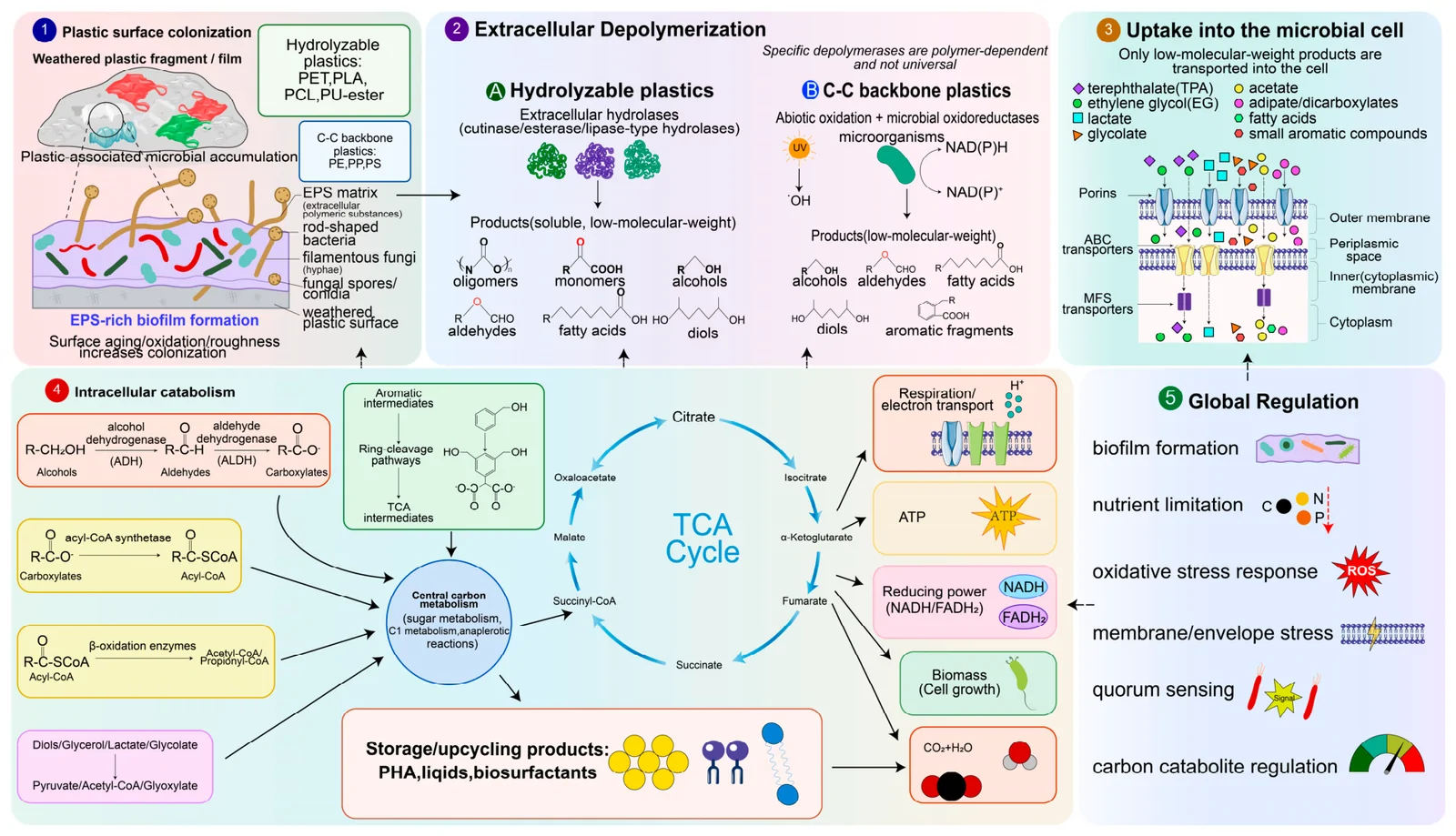
Microbial uptake, intracellular catabolic funneling, and metabolic fate of plastic-derived compounds. Microbial assimilation of plastic-derived carbon generally requires extracellular depolymerization or oxidative activation at the plastic–biofilm interface, which converts insoluble polymers into transportable low-molecular-weight products. After uptake through substrate-specific transport systems, these compounds are funneled into central metabolism through oxidation to carboxylates, acyl-CoA formation and β-oxidation, glyoxylate-related pathways, aromatic ring activation and cleavage, glycolysis, and the tricarboxylic acid (TCA) cycle. The resulting carbon flux can support respiration and ATP generation, biomass synthesis, mineralization to CO_2_, or biosynthesis of storage and value-added products such as polyhydroxyalkanoates (PHAs), lipids, biosurfactants, and organic acids.

Microbial plastic biodegradation is a multistep process controlled by polymer chemistry, surface accessibility, extracellular-enzyme activity, transport of soluble intermediates, intracellular catabolism, and physiological regulation (Figure 2). Because most synthetic polymers are insoluble, hydrophobic, and too large to enter microbial cells directly, biodegradation does not begin with intracellular metabolism. Instead, microbial utilization requires prior conversion of solid polymers into low-molecular-weight compounds at the polymer–biofilm interface. This section summarizes the key mechanistic steps of microbial plastic biodegradation, including abiotic surface activation, microbial colonization, extracellular depolymerization or oxidation, uptake of plastic-derived products, metabolic funneling, and regulatory constraints.

### 2.1. Polymer Aging and Bioavailability as Preconditions for Microbial Attack

The biodegradability of plastics is strongly constrained by polymer structure, crystallinity, molecular weight, hydrophobicity, additive composition, and surface chemistry [23]. After environmental release, plastics undergo photochemical oxidation, thermal oxidation, hydrolysis where applicable, mechanical abrasion, and chemical weathering. These processes increase surface roughness, generate cracks and pits, introduce carbonyl, hydroxyl, and carboxyl groups, and fragment larger debris into smaller particles [24]. However, fragmentation alone should not be interpreted as biodegradation. Its mechanistic significance is that it increases surface area and may expose oxidized or lower-molecular-weight regions that are more accessible to microbial attachment and enzymatic attack [10]. Thus, abiotic aging acts as a priming step that can improve polymer bioavailability, especially for highly recalcitrant carbon–carbon-backbone plastics such as PE, PP, and PS. In contrast, hydrolyzable polyesters such as PET, PLA, PCL, and polyester-type polyurethanes are more directly susceptible to enzymatic cleavage because their ester bonds provide chemically accessible targets for hydrolases [25].

### 2.2. Microbial Attachment and Plastisphere Biofilm Formation

Microbial degradation begins at the polymer surface [26]. Bacteria, fungi, microalgae, and mixed communities attach to plastics through hydrophobic interactions, electrostatic forces, extracellular polymeric substances, adhesins, and conditioning films derived from organic matter [27]. The resulting plastisphere is not only a microbial habitat but also a reaction interface where cells, enzymes, substrates, additives, and metabolites are spatially concentrated. Biofilm formation can enhance degradation by increasing residence time on the polymer surface, retaining extracellular enzymes near insoluble substrates, and enabling metabolic cooperation among community members [28]. For example, one population may hydrolyze or oxidatively activate the polymer surface, whereas other community members consume released oligomers, monomers, or inhibitory intermediates [29,30]. However, biofilm architecture can also impose diffusion limitations, generate oxygen or nutrient gradients, and intensify competition for accessible carbon sources [31]. Therefore, plastisphere formation should be viewed as a regulatory and catalytic microenvironment rather than merely surface colonization. However, microbial colonization should not be equated with true biodegradation, because surface attachment and biofilm development may occur without polymer depolymerization, assimilation of plastic-derived carbon, or mineralization [32].

### 2.3. Extracellular Depolymerization and Oxidative Chain Cleavage

The central bottleneck in microbial plastic biodegradation is extracellular conversion of insoluble polymers into soluble or cell-accessible compounds. For hydrolyzable plastics, extracellular or cell-surface-associated hydrolases, including cutinases, esterases, lipases, PETases, and polyurethane-active hydrolases, can adsorb to polymer surfaces and cleave ester bonds in polyesters and polyester-type polyurethanes; a smaller group of urethanases can further hydrolyze urethane/carbamate bonds under defined substrate conditions [33]. PET degradation proceeds through surface hydrolysis by PETase-like enzymes, releasing MHET as the major product together with TPA and BHET, followed by MHETase-mediated hydrolysis of MHET into TPA and ethylene glycol [34]. PLA hydrolysis can release lactic acid monomers, dimers, and higher oligomers, whereas PCL hydrolysis yields 6-hydroxyhexanoic acid and/or caprolactone-derived oligomers, depending on the enzyme and reaction conditions [35]. These reactions are controlled by enzyme adsorption, active-site accessibility, polymer chain mobility, crystallinity, temperature, water availability, and product inhibition.

For carbon–carbon-backbone plastics such as PE, PP, and PS, hydrolase-mediated chain cleavage is chemically unfavorable because these polymers lack readily hydrolyzable ester, amide, or urethane bonds. Their microbial transformation is therefore generally associated with oxidative activation, which introduces carbonyl or hydroxyl groups, promotes chain scission, and generates lower-molecular-weight products that are more accessible to microbial metabolism [36]. Abiotic photo- or thermo-oxidation and enzyme-mediated redox systems can introduce carbonyl groups, promote chain scission, and generate oxidized low-molecular-weight products [37]. Experimental studies have shown that laccase-mediated systems can oxidize PE and produce alcohols, aldehydes, and ketones [38], whereas manganese peroxidase and alkane hydroxylase systems have been implicated in PE or low-molecular-weight PE transformation [39,40]. For aromatic polymers such as polystyrene, oxygen-dependent transformation of styrene-derived intermediates may proceed through aromatic ring activation and cleavage or through phenylacetate-related catabolic routes, although direct evidence linking these pathways to complete PS mineralization remains limited [41,42]. These products can become substrates for downstream microbial metabolism, but evidence for complete mineralization is often limited and must be distinguished from surface erosion, mass loss, additive removal, or physical fragmentation. Therefore, rigorous assessment of biodegradation requires tracking polymer-derived carbon into soluble products, biomass, CO_2_, and specific metabolic intermediates.

### 2.4. Uptake and Intracellular Catabolic Funneling of Plastic-Derived Intermediates

Microorganisms generally assimilate plastic carbon only after extracellular depolymerization or oxidation has generated low-molecular-weight products [43]. These products enter cells through substrate-specific uptake systems, including TPA transporters such as TpiA/TpiB/TphC, MFS aromatic-acid symporters such as PcaK, HcnK, and VanK, outer-membrane porins or channels, and other transporters depending on substrate chemistry and host organism [44,45,46]. Once internalized, chemically diverse plastic-derived intermediates are funneled into a smaller number of central metabolic routes, as demonstrated by engineered PET-monomer assimilation and conversion systems in *Pseudomonas putida* and related hosts [47]. Aliphatic alcohols and aldehydes, including EG and oxidized PE-derived products, can be oxidized to carboxylates, whereas longer-chain carboxylates or fatty-acid-like intermediates can be processed through acyl-CoA-dependent and β-oxidation-related routes [30]. Diols, lactate, glycolate, and hydroxyacids can enter pyruvate, acetyl-CoA, glyoxylate, or tricarboxylic acid cycle metabolism [48]. Aromatic intermediates derived from PET, PS, or aromatic additives are generally metabolized through oxygenation and ring-cleavage pathways before entering central metabolism via protocatechuate, catechol, phenylacetate, or related intermediates [49,50].

The metabolic fate of plastic-derived carbon depends on substrate composition, pathway capacity, redox balance, nutrient availability, and the ability of host cells to assimilate or detoxify accumulated intermediates [50]. Carbon may be respired for ATP generation, incorporated into biomass, mineralized to CO_2_ and H_2_O, or redirected into storage and value-added products such as polyhydroxyalkanoates, lipids, biosurfactants, and organic acids [51]. Therefore, microbial plastic biodegradation should be evaluated not only by polymer mass loss but also by carbon flux: depolymerization products, uptake rates, intracellular intermediates, mineralization, and biomass or product formation [52].

### 2.5. Physiological Regulation, Rate-Limiting Steps, and Biotechnological Implications

Plastic biodegradation is regulated by nutrient availability, carbon catabolite repression, oxygen availability, oxidative stress, membrane or envelope stress, quorum sensing, and biofilm development [53]. These regulatory processes determine whether microorganisms invest in attachment, extracellular-enzyme secretion, transporter expression, stress tolerance, or growth on more accessible substrates. Nitrogen, phosphorus, and trace element limitation may restrict enzyme production and biomass formation even when polymer carbon is present [54]. Conversely, labile carbon sources may repress plastic-degradation pathways, although they can also support cometabolic oxidation of recalcitrant polymers [55]. Hydrophobic plastic surfaces, leached additives, oxidized fragments, metals, and coexisting pollutants can impose membrane, oxidative, and community-level stress, thereby reshaping plastic-associated microbiota and selecting for stress-tolerant taxa [56,57].

**Figure 3:**
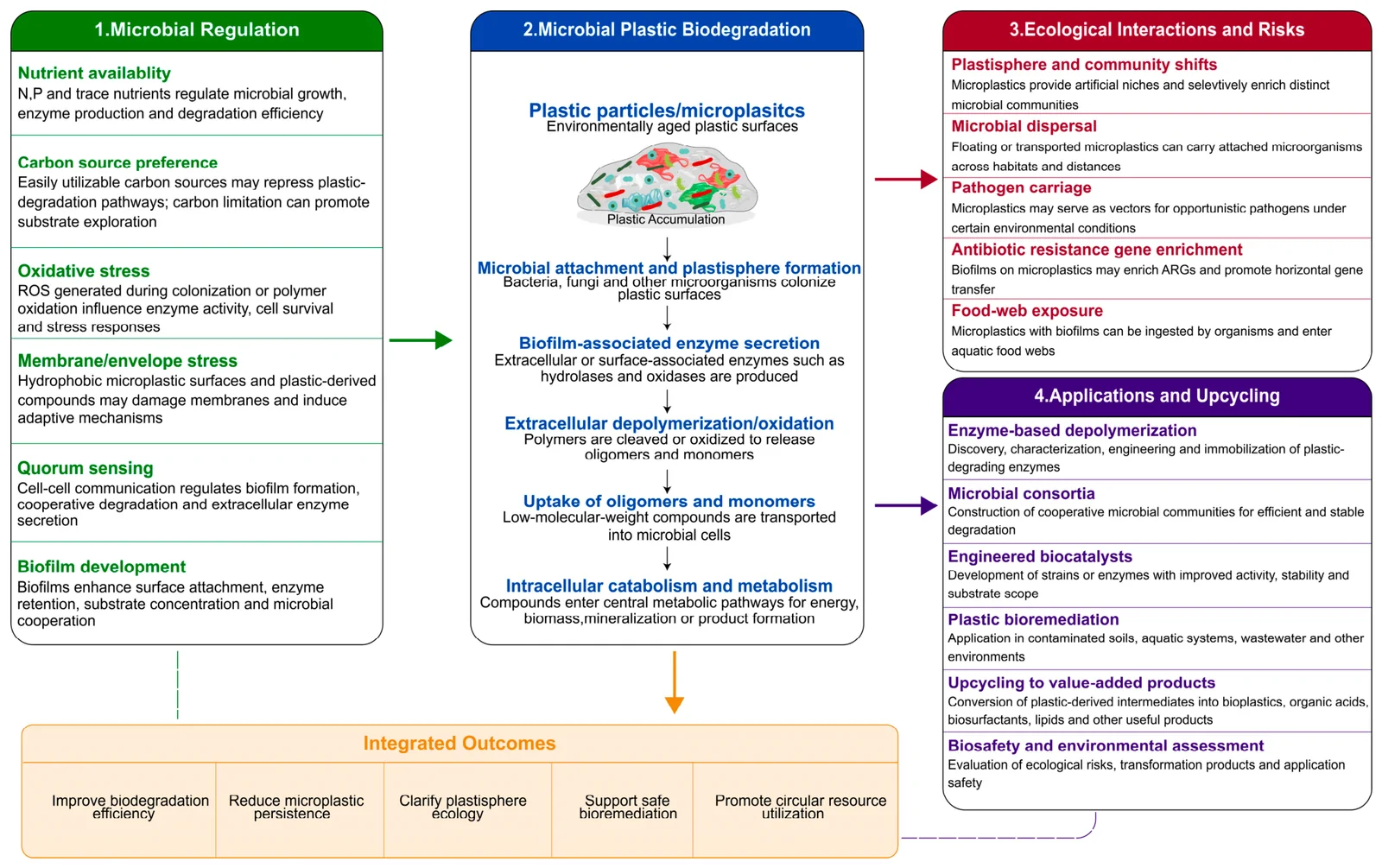
Regulatory factors, ecological interactions, and biotechnological applications of microbial plastic biodegradation. Physiological and environmental factors, including nutrient availability, carbon source preference, oxidative stress, membrane or envelope stress, quorum sensing, biofilm development, and coexisting pollutants, regulate microbial attachment, plastisphere formation, enzyme secretion, extracellular depolymerization or oxidation, substrate uptake, and intracellular metabolism. Plastics and microplastics can also serve as microbial habitats and contaminant carriers, contributing to community shifts, microbial dispersal, pathogen carriage, antibiotic resistance gene enrichment, and food-web exposure. Understanding these mechanisms can support enzyme-based depolymerization, microbial consortia, engineered biocatalysts, plastic bioremediation, and upcycling strategies aimed at improving degradation efficiency, reducing plastic persistence, and enabling safer circular resource use.

Mechanistically, the main rate-limiting steps differ among polymer types. For polyesters, enzyme adsorption, depolymerization efficiency, crystallinity, and product inhibition are often critical [58]. For PE, PP, and PS, oxidative activation and chain scission are usually the major constraints [41,59,60]. For all plastics, uptake capacity, catabolic pathway completeness, nutrient balance, and toxicity of intermediates influence whether partial transformation proceeds to assimilation or mineralization. These constraints are directly relevant to applications [51]. Enzyme engineering can improve substrate binding, thermostability, catalytic efficiency, and resistance to product inhibition [61]. Synthetic consortia can divide labor between depolymerization, intermediate detoxification, and product formation [30]. Engineered strains can channel plastic-derived carbon into bioproducts, but environmental deployment requires assessment of transformation products, gene-transfer risk, ecological disturbance, and containment [62]. Future research should therefore move beyond reporting plastic weight loss and instead integrate polymer chemistry, isotope tracing, omics-based pathway reconstruction, enzymology, metabolite analysis, and carbon-balance measurements to determine when microbial plastic degradation represents true assimilation, mineralization, or only superficial fragmentation. The regulatory factors, ecological interactions, and application-oriented implications of microbial plastic biodegradation are summarized in Figure 3.

### 2.6. The Most Promising Microorganisms for Future Research Based on Current Data

Current evidence does not support a universal taxonomic ranking of plastic-degrading microorganisms independent of polymer chemistry. A microorganism that is promising for an ester-containing polyester may be ineffective against a carbon–carbon-backbone polymer, and a strain capable of consuming released monomers may not be capable of initiating polymer depolymerization. Therefore, candidate microorganisms should be prioritized by matching their taxonomic and physiological characteristics to the rate-limiting step of the target polymer. The most important traits include extracellular or surface-associated enzyme secretion, stable attachment and biofilm formation, oxidative activation capacity, tolerance to polymer additives and degradation products, uptake of released oligomers or monomers, completeness of intracellular catabolic pathways, and the ability to redirect polymer-derived carbon into biomass, CO_2_, or value-added products.

For PET and other hydrolyzable polyesters, bacteria and fungi that produce extracellular cutinases, PETases, esterases, or lipases currently represent the most strongly supported groups. *Ideonella sakaiensis* remains an important whole-cell model because it couples PET depolymerization with assimilation of the resulting terephthalic acid and ethylene glycol through PETase- and MHETase-associated reactions [34]. *Thermobifida* species are promising actinobacterial enzyme sources because their cutinases can hydrolyze PET surfaces, while *Fusarium solani* and *Candida antarctica* provide fungal or yeast-derived cutinases and lipases capable of hydrolyzing polyester substrates [33,58]. In parallel, metabolically versatile Proteobacteria, including *Pseudomonas* and *Comamonas*, are particularly promising as assimilation and upcycling hosts because they possess transport and catabolic systems for terephthalate, ethylene glycol, aromatic acids, and related intermediates [44,45,46,47,62]. These organisms may not always be the most efficient primary polymer depolymerizers, but they are valuable downstream hosts for coupling extracellular hydrolysis with intracellular conversion.

For polyethylene, polypropylene, and polystyrene, the evidence is less conclusive, and greater priority should be given to hydrocarbon-degrading, biofilm-forming, and oxidatively active microorganisms. Actinobacteria such as *Rhodococcus* are promising because of their hydrophobic surface attachment, persistent biofilm formation, laccase-associated oxidation, and capacity to metabolize hydrocarbon-like intermediates [36,43,63,64]. *Pseudomonas* strains containing alkane hydroxylase systems may also be relevant for the transformation of low-molecular-weight or previously oxidized polyethylene products [40]. *Bacillus*- and *Brevibacillus*-related bacteria are attractive because of their environmental persistence, enzyme-secretion capacity, and reported activity in single-strain or consortium-based polyolefin experiments [29,60]. Among fungi, *Zalerion maritimum* and other extracellular oxidative-enzyme producers warrant further investigation because fungal systems can persist on nutrient-poor plastic surfaces and have been associated with chemical changes in polyethylene microplastics [39,65]. However, evidence for these resistant polymers remains substantially weaker than that for enzymatic PET hydrolysis, and claims of complete degradation should remain conditional until polymer-derived carbon has been traced into biomass, metabolites, or CO_2_.

Microbial consortia may be more promising than individual isolates when complete degradation requires several physiologically distinct functions. A primary colonizer or extracellular-enzyme producer may initiate surface oxidation or depolymerization, while a second population consumes oligomers or monomers, removes inhibitory intermediates, or converts the resulting carbon into a target product. Consortia containing *Pseudomonas* and *Bacillus* species have demonstrated synergistic effects on PET-associated transformation, and engineered division-of-labor systems have shown that depolymerization, intermediate assimilation, and product biosynthesis can be distributed among different populations [29,30]. Nevertheless, consortium stability, competition for alternative substrates, reproducibility, and preservation of functional interactions under environmental conditions remain important limitations.

Conversely, no complete microbial phylum should currently be classified as intrinsically unsuitable for plastic degradation. The weakest candidates are instead defined by insufficient physiological and experimental evidence. Generalist microorganisms detected only on plastic surfaces, strains that grow only in the presence of added labile carbon, organisms that utilize leached additives rather than polymer-derived carbon, and isolates supported solely by weight loss, SEM images, or FTIR changes should not be regarded as confirmed primary degraders. A stepwise screening hierarchy is therefore recommended: demonstration of reproducible surface-associated activity with appropriate abiotic controls; detection of molecular-weight reduction and defined oligomers or monomers; growth or product formation from polymer-derived carbon; isotope-based evidence of carbon incorporation or mineralization; and genetic or enzymatic validation of the responsible pathways [32,43,52,66,67].

On the basis of current evidence, the most promising systematic groups include extracellular-enzyme-producing Actinobacteria, metabolically versatile Proteobacteria, environmentally robust *Bacillus*- and *Brevibacillus*-related bacteria, enzyme-secreting fungi and yeasts, and stable microbial consortia. However, physiological capability and polymer-specific evidence are more informative than taxonomy alone. Future discovery programs should therefore combine cultivation, metagenomics, functional screening, enzyme characterization, stable-isotope tracing, and polymer-chemistry measurements rather than selecting candidates solely because they are abundant members of the plastisphere.

## 3. Materials and Methods

### 3.1. Data Source and Literature Search

This study used the Web of Science Core Collection (WoSCC) as the sole data source for bibliometric data retrieval. To obtain publications specifically related to microbial plastic biodegradation, a field-specific proximity search was conducted in the title, abstract, and author keyword fields. The search strategy combined three groups of terms: plastic-related terms, biodegradation-related terms, and microbial or enzymatic degradation-related terms. Because the full search strategy was relatively long and complex, the complete query was provided in Supplementary File S1.

The search was conducted on 14 April 2026, and the publication period was limited to 2000–2025. Only English-language publications categorized as Article or Review were included to ensure consistency and comparability of the dataset. All retrieved records were independently screened by two reviewers based on titles, abstracts, author keywords, and, when necessary, full bibliographic information. Publications were retained when microbial, fungal, bacterial, enzymatic, or microbial consortium-mediated plastic degradation was a central topic. Records were excluded when they mainly focused on non-biological plastic aging, purely chemical degradation, mechanical recycling, material synthesis without biodegradation assessment, or biodegradable polymer systems not directly related to microbial or enzymatic degradation.

Disagreements between the two reviewers were resolved through discussion and rechecking of the corresponding records. Cases that could not be resolved by consensus were evaluated by a third reviewer. After manual screening, 2959 valid publications were retained for subsequent bibliometric analysis. All eligible records were exported from the WoSCC as plain text files in the “Full Record and Cited References” format and used as the data basis for bibliometric and visualization analyses.

### 3.2. Bibliometric Analysis and Visualization

The retrieved bibliographic records were used for bibliometric analysis, collaboration network construction, knowledge structure identification, and emerging research frontier detection. Before keyword and network analyses, the bibliographic data were checked to reduce obvious inconsistencies. Synonymous keywords, spelling variants, and singular/plural forms were manually merged where appropriate. The bibliometrix R package version 4.5.0 was used to conduct Bradford’s law analysis and identify core source journals [68,69]. It was also used to generate keyword temporal trend charts. Journal Impact Factor values and JCR quartile rankings were obtained from the 2025 Journal Citation Reports released by Clarivate. VOSviewer 1.6.20 was used to construct the author collaboration network and keyword co-occurrence network. [70]. These analyses were used to identify productive author groups, collaborative patterns, and major keyword associations in microbial plastic biodegradation research.

In addition, CiteSpace 7.0.R0 was used to analyze temporal evolution and emerging research hotspots [71]. The time span was set from 2000 to 2025, with one year per slice. Keywords were selected as the node type for burst detection, and the g-index with k = 25 was used as the node selection criterion. Pathfinder, sliced-network pruning, and merged-network pruning were applied to improve network clarity and interpretability. CiteSpace was also used to generate the journal dual-map overlay, which was applied to reveal citation flows between citing and cited journal disciplines. SCImago Graphica Beta 1.0.51 was used to visualize the geographical distribution map and institutional collaboration network [72]. These maps were used to display country/region-level research distribution and collaboration patterns among major institutions. Microsoft Excel 2024 was used for data organization, descriptive statistics, and figure preparation.

## 4. Results and Discussion

### 4.1. General Overview and Temporal Trends of the Publications

**Figure 4:**
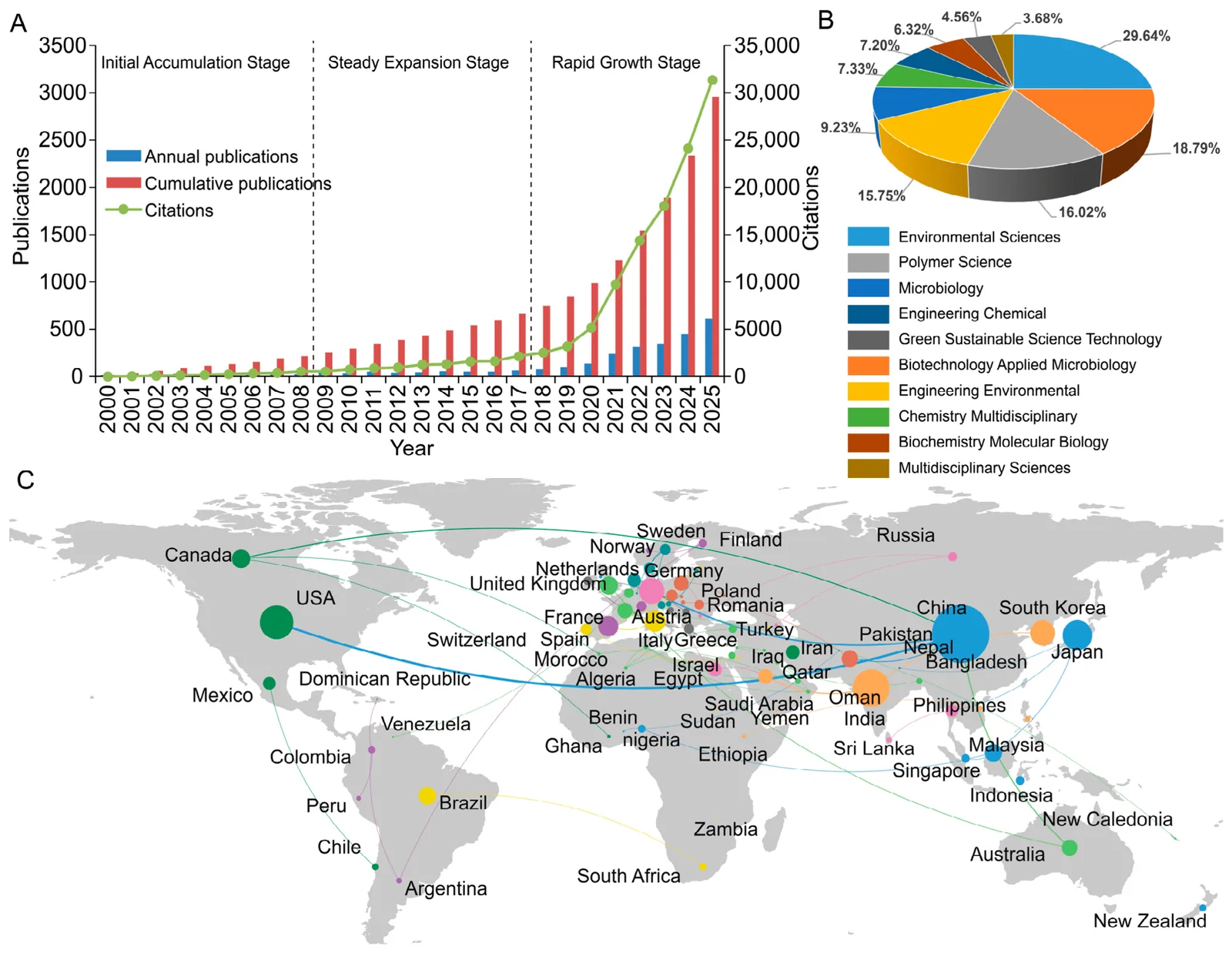
Temporal publication trends, subject category distribution, and country/region collaboration network of microbial plastic biodegradation research from 2000 to 2025. (**A**) Annual publications, cumulative publications, and citation trends. (**B**) Distribution of major Web of Science subject categories. As individual publications may be assigned to multiple Web of Science subject categories, the percentages do not sum to 100%. (**C**) Country/region distribution and international collaboration network. Node size indicates publication output, and connecting lines indicate collaborative relationships among countries/regions.

As shown in Figure 4A, annual publications on microbial plastic biodegradation increased from 20 in 2000 to 617 in 2025, representing an approximately 30.9-fold increase, while the cumulative number of publications reached 2959 and citations reached 31,373. Based on annual publication output, the development of the field can be divided into three stages: an initial accumulation stage from 2000 to 2008, a steady expansion stage from 2009 to 2017, and a rapid growth stage from 2018 to 2025. Only 664 publications had accumulated by 2017, whereas 2295 publications were published between 2018 and 2025, accounting for approximately 77.6% of the complete dataset. Annual output also increased from 80 publications in 2018 to 617 publications in 2025, corresponding to a 7.7-fold increase within eight years. This concentration of research output after 2018 is scientifically important because it marks the transition of microbial plastic biodegradation from a relatively dispersed research topic into a rapidly expanding interdisciplinary field. The simultaneous acceleration of citations, particularly after 2020, further indicates that the recent increase reflects not only greater publication volume but also growing uptake and integration of the resulting knowledge.

This rapid growth after 2018 likely reflects the convergence of scientific, environmental concern, and methodological advances rather than a simple increase in publication volume. On the scientific side, research on microbial and enzymatic plastic degradation expanded from strain screening and surface deterioration observations to more mechanism-oriented studies [19]. Among different plastics, PET degradation received particular attention because PETase- and MHETase-associated systems provided a relatively clear enzymatic model for plastic depolymerization, enzyme engineering, and downstream conversion of plastic-derived monomers [34,73]. Subsequent advances in engineered plastic-degrading enzymes further strengthened the link between microbial biodegradation and plastic recycling or upcycling [61]. At the same time, studies on other polymers, including PE, PP, PS, polyurethane, and biodegradable polyesters, also increased, although their degradation mechanisms and efficiency remain more variable and polymer-dependent [10]. On the environmental and policy side, increasing concern about microplastic pollution, plastic persistence, and circular resource management promoted microbial degradation as a potential mitigation strategy [74,75,76].

The subject category distribution also reflects this research orientation (Figure 4B). Environmental sciences accounted for the largest proportion, followed by polymer science, microbiology, and engineering chemistry. This distribution is consistent with the core logic of the field: environmental pollution provides the problem background, polymer science explains the material properties that limit degradation, microbiology provides the degrading organisms and enzymes, and chemical engineering supports process optimization and potential recycling applications.

### 4.2. Scientific Collaboration Network

#### 4.2.1. Country-Level Collaboration

Figure 4C and Table 1 summarize the country/region-level publication output and collaboration network in microbial plastic biodegradation research. China ranked first with 892 publications, accounting for approximately 30% of the total publications, followed by India with 369 publications and the United States with 301 publications. The top contributing countries were mainly distributed in Asia, North America, and Europe, indicating that this field has developed into an international research topic rather than being concentrated in a single region.

China’s leading publication output may be partly associated with its strong research demand in plastic pollution control, environmental remediation, biodegradable materials, and resource recycling. In recent years, China has strengthened plastic pollution governance in response to increasing plastic consumption associated with e-commerce, food delivery, and other rapidly growing sectors [77]; in 2020, the National Development and Reform Commission and the Ministry of Ecology and Environment issued policy measures to further control plastic pollution [75], and the 2021–2025 action plan further emphasized whole-chain plastic pollution control [78]. These policies and environmental pressures may have stimulated research on microbial degradation, enzymatic depolymerization, microplastic transformation, and biotechnological treatment strategies. In addition, China’s broad research base in environmental science, polymer materials, microbiology, and biotechnology may also contribute to its high publication output [79]; similar bibliometric studies on microplastic pollution have also reported China as one of the most productive countries in related fields [80].

Publication output and betweenness centrality represented two distinct dimensions of national contribution. Although China and India ranked first and second in publication output, with 892 and 369 publications, respectively, their betweenness centrality values were only 0.15 and 0.09. By contrast, the United States had the highest betweenness centrality of 0.83 despite ranking third in publication output, while Italy showed a similarly disproportionate bridging role, with a centrality of 0.55 despite ranking seventh in output. Germany and the United Kingdom also exhibited relatively high centrality values of 0.26 and 0.24, respectively. This divergence demonstrates that high productivity does not necessarily correspond to control over international knowledge exchange. Scientifically, the network appears to depend on a limited number of bridging countries that connect otherwise regionally concentrated research clusters. Such dependence may restrict the international transfer of strains, enzyme resources, standardized degradation protocols, and environmentally validated datasets. Future collaboration should therefore connect high-output research systems in Asia with the strong bridging networks of the United States and Europe, while increasing the participation of currently underrepresented regions. International consortia would be particularly valuable for interlaboratory validation of plastic-degrading microorganisms, carbon-balance measurements, reference-material development, and field-scale assessment under different climatic and environmental conditions.

#### 4.2.2. Institution Collaboration

**Figure 5:**
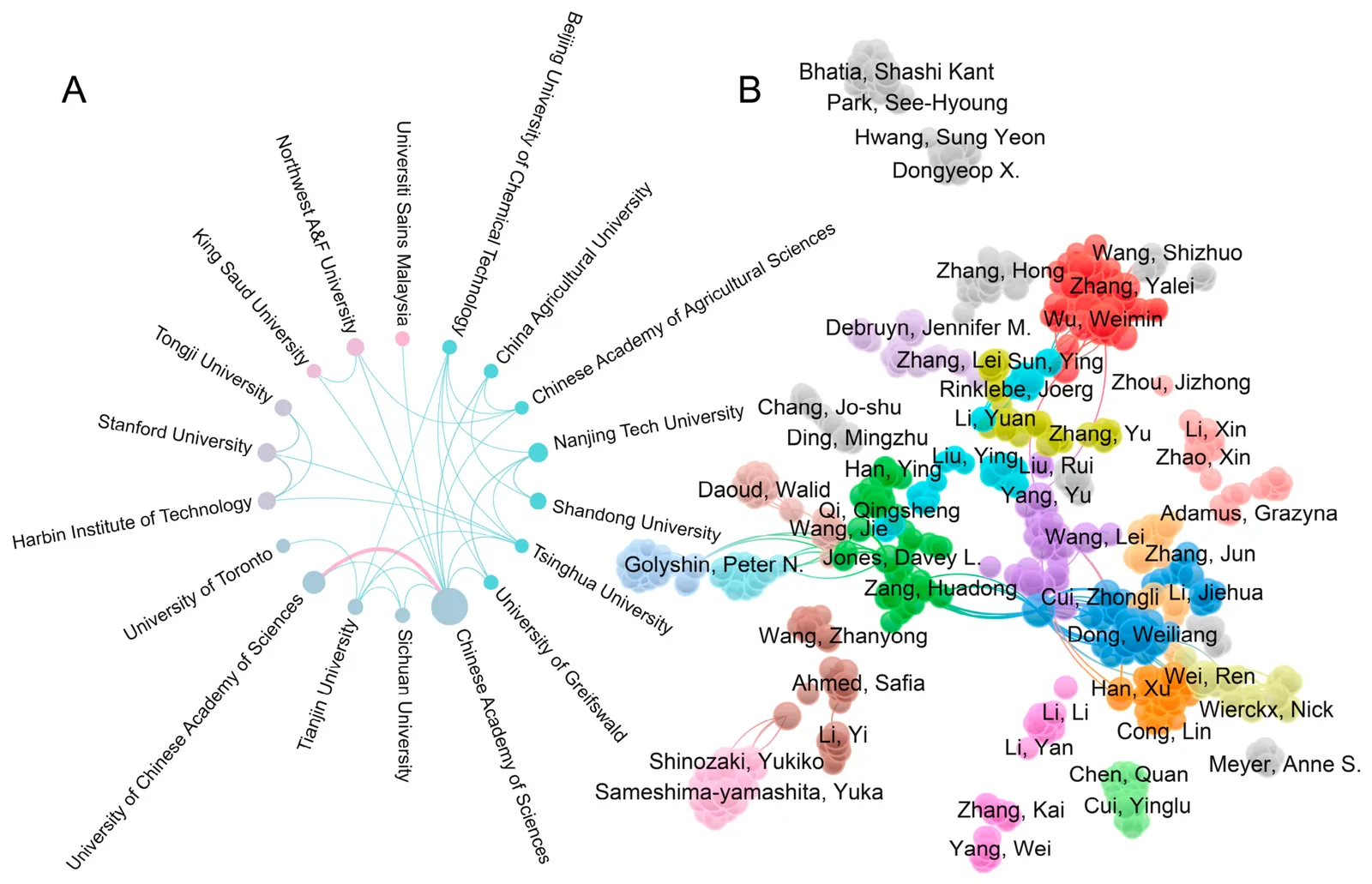
Collaboration patterns among institutions and authors in microbial plastic biodegradation research. (**A**) Institutional collaboration network. (**B**) Author collaboration network. Nodes represent institutions or authors, and node size indicates their relative contribution or weight in the collaboration network. Connecting lines indicate collaborative relationships, with thicker lines representing stronger collaboration strength. Different colors represent distinct collaboration clusters.

Institution-level analysis showed that institutional contributions to microbial plastic biodegradation research were highly concentrated in China (Table 1 and Figure 5A). The Chinese Academy of Sciences ranked first with 144 publications and 6770 citations, showing a clear leading position in both publication output and citation impact. The University of Chinese Academy of Sciences ranked second with 56 publications and 2250 citations, followed by the National Institute of Advanced Industrial Science and Technology in Japan with 43 publications and 3314 citations. Among the top 10 institutions, eight were from China, indicating that Chinese research institutions formed the main institutional base of this field.

Although publication output was concentrated in Chinese institutions, citation impact was not determined only by the number of publications. Stanford University ranked fifth in publication output with 38 publications but received 4496 citations, indicating strong academic influence. Similarly, the National Institute of Advanced Industrial Science and Technology showed a high citation count despite a moderate publication output. These results suggest that institutional influence in microbial plastic biodegradation research depends not only on productivity, but also on the quality, visibility, and citation performance of representative studies.

The institutional collaboration network in Figure 5A further shows that the Chinese Academy of Sciences occupied a central position and had collaborative links with several domestic and international institutions. However, the overall network was still relatively sparse, with collaborations mainly occurring around a limited number of productive institutions. This pattern suggests that institutional collaboration in microbial plastic biodegradation research has formed several local or regional clusters, but a highly integrated global collaboration network has not yet been fully established.

#### 4.2.3. Author Collaboration

Author-level analysis showed that several productive research groups have emerged in microbial plastic biodegradation research (Table 1 and Figure 5B). Weiliang Dong ranked first with 36 publications, followed by Wei-Min Wu and Ren Wei with 33 and 32 publications, respectively. However, publication output and citation impact were not completely consistent. Wei-Min Wu had the highest citation count with 4234 citations, far exceeding that of the most productive author, Weiliang Dong, who received 838 citations. This indicates that author influence in this field depends not only on publication quantity, but also on the citation performance and visibility of representative studies.

The country’s distribution of highly productive authors was relatively concentrated. Authors from Japan accounted for half of the top 10, while China contributed three authors, suggesting that author-level productivity was mainly concentrated in several active national research groups. The author collaboration network in Figure 5B further shows multiple distinct collaborative clusters. Although several stable teams have formed, the links among different clusters remain limited. This suggests that author collaboration in microbial plastic biodegradation research is still relatively fragmented, and broader cross-team cooperation has not yet been fully established.

### 4.3. Journal Distribution and Core Journal Analysis

**Figure 6:**
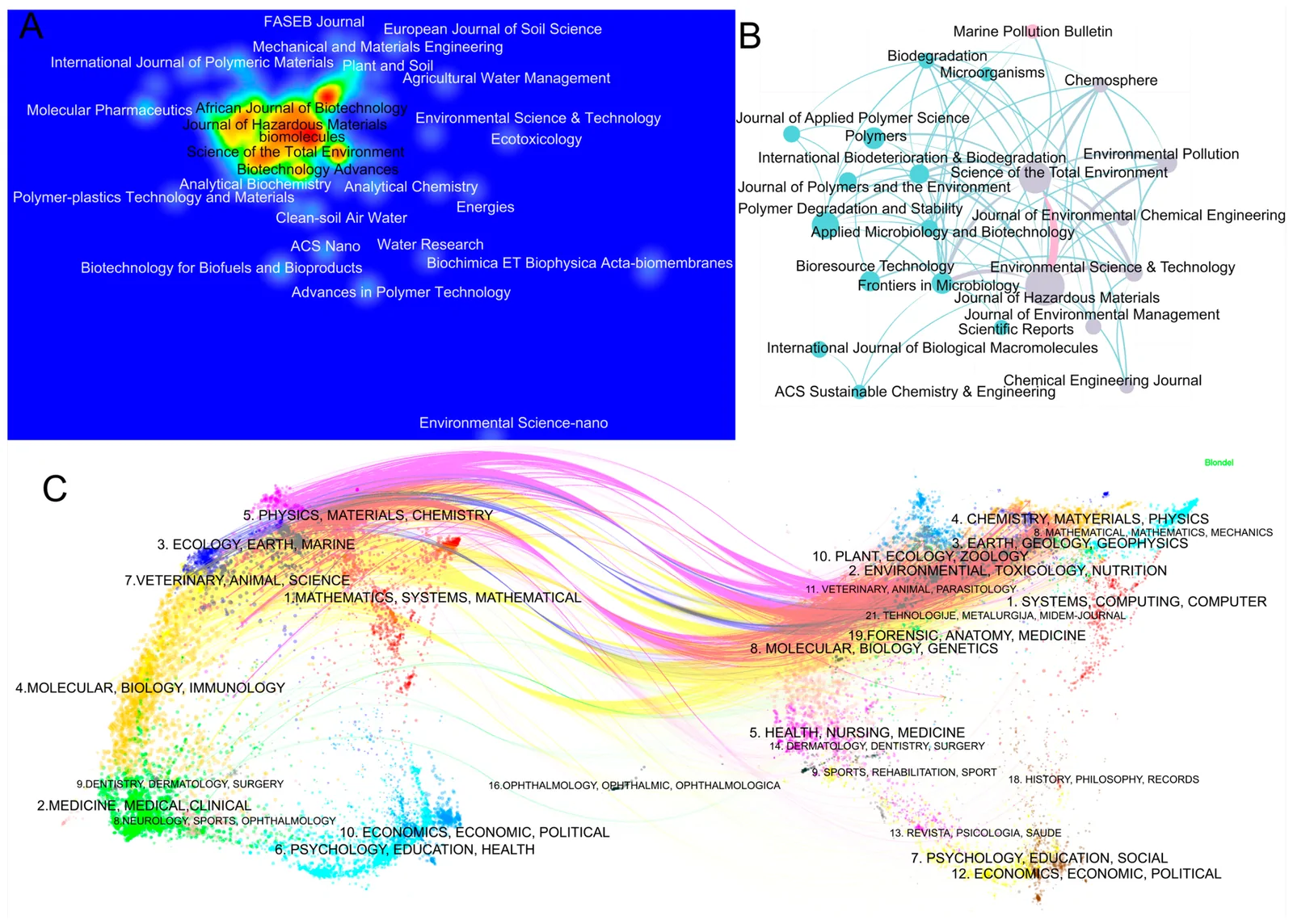
Journal distribution and citation relationships in microbial plastic biodegradation research. (**A**) Heatmap of source journals with at least five publications, where warmer colors indicate higher publication density. (**B**) Journal citation network, where nodes represent journals, node size reflects journal weight, and connecting lines indicate citation relationships. (**C**) Dual-map overlay showing citation flows from citing journal disciplines on the left to cited journal disciplines on the right; colored curves represent major citation paths.

Journal distribution analysis showed that microbial plastic biodegradation research was mainly published in journals related to environmental science, polymer degradation, microbiology, and biotechnology (Table 2 and Figure 6). Among the top 15 journals, *Journal of Hazardous Materials* ranked first with 193 publications, followed by *Science of the Total Environment* with 129 publications and *Polymer Degradation and Stability* with 92 publications. This pattern indicates that the field is not limited to polymer degradation itself, but is strongly connected with environmental pollution, hazardous materials, and plastic waste management.

The journal profile also showed relatively high academic visibility (Table 2). Among the top 15 journals, most were ranked in JCR Q1 in 2025. High-impact journals such as *Journal of Hazardous Materials*, *Environmental Science & Technology*, *Bioresource Technology*, *International Journal of Biological Macromolecules*, and *Journal of Environmental Management* indicate that studies on microbial plastic biodegradation have been published not only in specialized biodegradation or polymer journals, but also in broader environmental and biotechnology journals. This suggests that the topic has gained attention across both material-oriented and environment-oriented research communities.

The source-journal map in Figure 6A further shows that publication output was concentrated in a limited number of journals, with *Journal of Hazardous Materials*, *Science of the Total Environment*, *Polymer Degradation and Stability*, *Polymers*, and *Frontiers in Microbiology* forming major publication outlets. The journal citation network in Figure 6B shows close citation relationships among environmental science journals, polymer-related journals, and biodegradation or biotechnology journals. This indicates that knowledge exchange in this field occurs mainly between studies focusing on environmental pollution, polymer properties, microbial degradation, and applied biotechnological treatment.

The dual-map overlay in Figure 6C further supports this interdisciplinary structure. The main citation flows connected journals in ecology, environmental science, materials science, chemistry, and microbiology with cited journals in environmental toxicology, chemistry, materials science, molecular biology, and applied microbiology. This pattern suggests that microbial plastic biodegradation research draws on both material-level knowledge, such as polymer structure and degradation behavior, and biological knowledge, such as microbial metabolism and enzymatic depolymerization.

### 4.4. Analysis of Highly Cited Publications

Table 3 summarizes the top 10 most-cited publications representing the intellectual foundations of plastic biodegradation and biodegradable polymer research. The most-cited publication was the review by Shah et al., published in Biotechnology Advances in 2008, with 1907 citations [81], followed by the engineered PET depolymerase study by Tournier et al., published in Nature in 2020, with 1665 citations [61]. The review Biodegradability of Plastics by Tokiwa et al. received 1207 citations [82]. These citation patterns reveal two complementary forms of scientific influence. Highly cited reviews established the conceptual and methodological framework for evaluating plastic biodegradability, whereas highly cited experimental studies provided tractable enzyme systems capable of connecting polymer depolymerization with monomer recovery and recycling. Thus, citation impact in this field is concentrated around publications that either integrated fragmented degradation evidence into a coherent framework or introduced experimentally reproducible systems with clear mechanistic and application potential. The citation structure therefore reflects the broader transition of the field from determining whether plastic deterioration can occur toward understanding, engineering, and controlling microbial or enzymatic depolymerization.

The first important knowledge component is the general understanding of plastic biodegradation. Reviews on biological degradation of plastics, plastic biodegradability, and microbial and enzymatic degradation of synthetic plastics provided the conceptual framework for this field by summarizing microbial participation, enzymatic reactions, polymer structure effects, degradation pathways, and environmental factors [81,82,86]. These publications helped integrate scattered evidence from strain screening, polymer degradation tests, and biodegradability evaluation into a more systematic understanding of plastic degradation.

The second component is biodegradable polymer and bioplastic research. Highly cited studies on poly(vinyl alcohol)-based materials, bacterial polyesters, PHA bioplastics, biodegradable mulches, and bioplastics degradation in natural environments indicate that polymer type, chemical structure, crystallinity, application scenario, and environmental exposure conditions strongly influence degradation performance [83,84,85,88,90]. These studies are not all narrowly focused on microbial degradation, but they provide important material-level knowledge for evaluating whether a polymer can be degraded and under what conditions degradation may occur.

The third component is mechanism-oriented research on resistant plastics and enzymatic depolymerization. The study on polyethylene biodegradation by bacteria from waxworm guts provided biological evidence for the possible degradation of a highly persistent polyolefin plastic [87]. In contrast, the engineered PET depolymerase study showed a clearer enzymatic route for PET depolymerization and recycling, linking plastic biodegradation research with enzyme engineering and resource recovery [61]. Together, these studies reflect two major directions in the field: exploring microbial degradation of environmentally persistent plastics and developing enzyme-based strategies for more controllable plastic depolymerization.

### 4.5. Keyword Co-Occurrence and Research Hotspot Analysis

**Figure 7:**
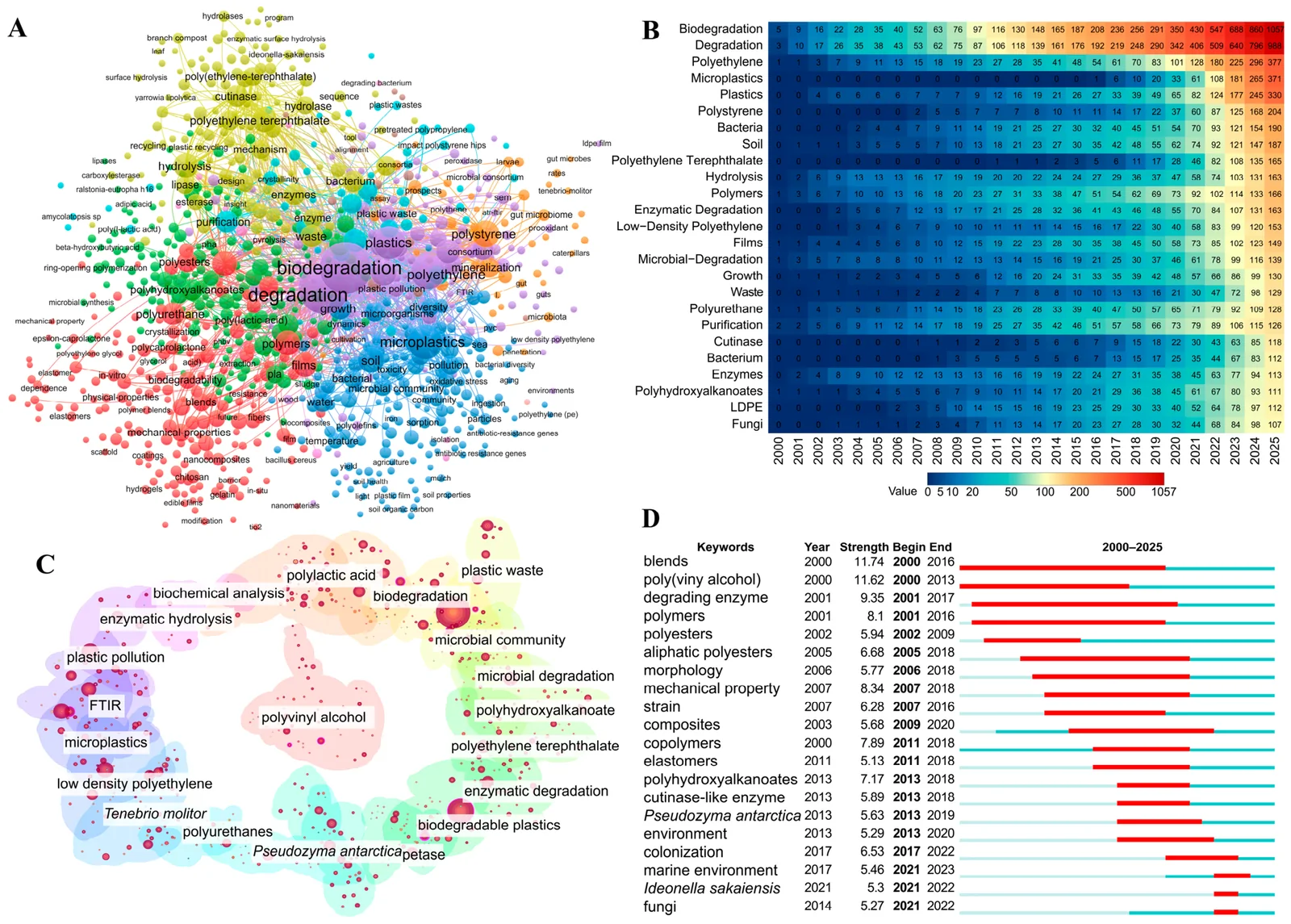
Keyword analysis of microbial plastic biodegradation research from 2000 to 2025. (**A**) Keyword co-occurrence network, where nodes represent keywords, node size indicates keyword frequency, connecting lines indicate co-occurrence relationships, and colors represent keyword clusters. (**B**) Temporal heatmap of high-frequency keywords, with warmer colors indicating higher annual frequencies. (**C**) Keyword clustering map, where colored regions represent different thematic clusters. (**D**) Top 20 keywords with the strongest citation bursts; red bars indicate burst periods, and blue-green lines represent the full study period.

Keywords provide a direct reflection of the major research themes and their evolution in microbial plastic biodegradation research. The keyword co-occurrence network showed a clear thematic structure, with high-frequency terms such as “biodegradation”, “degradation”, “microplastics”, “plastics”, “polyethylene”, “polyethylene terephthalate”, “polyurethane”, “polystyrene”, “hydrolysis”, “enzyme”, “bacteria” and “strain” occupying central positions in the network (Figure 7A). These keywords indicate that the field has long focused on three closely related issues: degradation behavior of plastics and microplastics [91], polymer-specific biodegradation [26], and the roles of microorganisms and enzymes in polymer depolymerization [89]. In particular, the presence of polymer-specific terms such as “polyethylene” and “polyethylene terephthalate” suggests that both resistant polyolefin plastics and hydrolyzable polyester plastics have been major research objects [92], while “enzyme”, “hydrolysis”, “bacteria” and “strain” reflect the central role of microbial screening and enzymatic degradation mechanisms in this field [25].

The heatmap further showed that “biodegradation” and “degradation” remained the most frequent terms throughout the study period, with their frequencies increasing sharply after 2020 and reaching the highest values in 2025 (Figure 7B). This increase is consistent with the growing concern about plastic persistence, microplastic pollution, and the need for biological treatment strategies [93]. In addition, keywords such as “microplastics”, “plastics”, “polyethylene”, “polyethylene terephthalate”, “enzymatic degradation”, “microbial degradation”, “cutinase”, “bacterium”, “enzymes” and “fungi” increased markedly in recent years. These changes suggest that research attention has gradually shifted from general biodegradability evaluation toward polymer-specific degradation, microbial and enzymatic mechanisms, and environmental plastic pollution. In particular, the increase in “polyethylene” and “polyethylene terephthalate” reflects sustained interest in both resistant polyolefin plastics and hydrolyzable polyester plastics [94,95], while “enzymatic degradation”, “cutinase”, “enzymes”, and “fungi” indicate the growing importance of enzyme-mediated depolymerization and microbial degradation systems [25,53].

Keyword clustering analysis further revealed the knowledge structure of this field (Figure 7C). The clusters can be broadly grouped into three thematic directions. The first direction is polymer materials and biodegradability evaluation, represented by terms such as “polyvinyl alcohol”, “polyhydroxyalkanoate”, “polylactic acid”, “biodegradable plastics”, “polyurethane”, and “low density polyethylene.” This cluster indicates that polymer type and material properties remain the starting point of degradation research. Biodegradable polymers and conventional plastics differ greatly in chemical structure, crystallinity, hydrophobicity, and hydrolyzable bonds, which directly affect microbial colonization, enzyme accessibility, and degradation efficiency [65]. The second direction is microbial and enzymatic degradation, represented by “enzymatic degradation”, “enzymatic hydrolysis”, “microbial degradation”, “microbial community”, “PETase”, and “*Pseudozyma antarctica*”. Compared with the material-oriented cluster, this cluster reflects a shift from asking whether plastics can be degraded to explaining how biological degradation occurs. In this context, plastic-degrading strains, microbial communities, extracellular enzymes, and depolymerization reactions are central for understanding the rate-limiting steps of plastic biodegradation [53]. The appearance of PETase-related terms also suggests that enzymatic depolymerization has become an important mechanistic entry point, especially for polyester plastics with hydrolyzable ester bonds [96]. The third direction is environmental occurrence and degradation assessment, represented by “microplastics”, “plastic pollution”, “plastic waste”, “FTIR”, and “marine environment”. This cluster shows that microbial plastic biodegradation research is increasingly connected with real pollution scenarios rather than only laboratory degradation tests. Keywords related to FTIR and environmental matrices indicate that researchers are paying more attention to chemical bond changes, surface deterioration, and degradation behavior under complex environmental conditions [34,97]. This is important because degradation observed under controlled laboratory conditions may not directly translate to soil, freshwater, marine, composting, or waste-treatment systems.

The keyword burst analysis revealed a clear temporal shift in research hotspots from 2000 to 2025 (Figure 7D). Early burst keywords were mainly material-oriented, including “blends” (strength = 11.74, 2000–2016), “poly(vinyl alcohol)” (strength = 11.62, 2000–2013), “polymers” (strength = 8.10, 2001–2016), and “polyesters” (strength = 5.94, 2002–2009). These terms indicate that early studies were largely rooted in polymer science and biodegradability evaluation. For example, experimental studies on PVA and PVA-based blends evaluated biodegradation through microbial culture, activated sludge, soil burial, landfill leachate exposure, weight loss, FTIR changes, SEM observation, and changes in polymer structure or material properties [98]. Similarly, studies on biodegradable polyesters such as poly(ε-caprolactone) focused on microbial attack, surface erosion, mass loss, and changes in morphology or mechanical behavior [99]. Therefore, the early hotspot was not microbial degradation mechanisms in a strict sense, but whether specific polymer materials could undergo measurable deterioration or biodegradation under biological or environmental exposure.

During the middle period, burst keywords such as “aliphatic polyesters” (strength = 6.68, 2005–2018), “morphology” (strength = 5.77, 2006–2018), “mechanical property” (strength = 8.34, 2007–2018), “composites” (strength = 5.68, 2009–2020), “copolymers” (strength = 7.89, 2011–2018), and “polyhydroxyalkanoates” (strength = 7.17, 2013–2018) suggest that the field began to pay more attention to the relationship between polymer structure and degradation behavior. PCL/PLA-based blend films showed selective lipase-catalyzed degradation of the PCL component, accompanied by changes in blend morphology [100]. Annealed PCL films also exhibited morphology-dependent enzymatic degradation, indicating that crystallinity and surface structure can influence enzyme accessibility [101]. In addition, comparative enzymatic degradation of common aliphatic bio-polyesters showed that different hydrolytic enzymes, including lipases and cutinase, display different degradation efficiencies toward polyester substrates [102]. Studies on PHA/wood composites further showed that soil burial degradation is accompanied by changes in mechanical strength, chemical composition, and morphology [103]. These findings indicate that microbial or enzymatic degradation is strongly constrained by material properties, including crystallinity, surface morphology, chemical bonds, additives, copolymer composition, and composite structure.

More recent burst keywords were more closely associated with microbial and enzymatic degradation mechanisms. The long burst of “degrading enzyme” (strength = 9.35, 2001–2017) indicates that enzymatic degradation has been a persistent topic rather than a newly emerging one. However, the meaning of this topic has changed over time. Earlier enzymatic studies were often linked to broad degradation tests of biodegradable polyesters, whereas recent studies increasingly focus on defined enzymes, catalytic mechanisms, protein engineering, and depolymerization products. The discovery of *Ideonella sakaiensis* 201-F6 was a representative turning point because this bacterium can use PET as a major carbon and energy source and produces PETase and MHETase, which hydrolyze PET and its intermediate MHET into terephthalic acid and ethylene glycol [34]. Subsequent structural and biochemical studies further clarified the catalytic basis of PETase, including its active-site architecture, substrate-binding features, and relationship with cutinase-like hydrolases [104,105]. In addition, studies on PETase/MHETase synergy and engineered PET hydrolases showed that enzymatic plastic degradation could be linked with controlled depolymerization, monomer recovery, and PET recycling [61,106]. Therefore, PET degradation has become one of the clearest mechanistic models in microbial plastic biodegradation research.

The burst of “cutinase-like enzyme” (strength = 5.89, 2013–2018) also reflects the increasing importance of polyester-hydrolyzing enzymes. Cutinases, PETases, lipases, esterases, and related hydrolases are attractive because they directly connect polymer depolymerization with measurable products. In recent years, experimental studies have shifted from enzyme discovery to enzyme engineering and product-oriented depolymerization. For example, Tournier et al. reported an engineered PET depolymerase that achieved high PET depolymerization into monomers, linking enzymatic degradation directly with PET recycling [61]. Later, FAST-PETase demonstrated that machine learning-guided enzyme engineering could depolymerize a wide range of untreated post-consumer PET products within one week and support closed-loop PET recycling [106]. More recently, TurboPETase further showed that computational redesign can markedly improve PET hydrolase performance, achieving nearly complete PET depolymerization under high-solids conditions [107]. These experimental advances explain why enzyme-related keywords became more mechanistic and application-oriented in the later stage.

At the same time, the burst keywords also show that the field is not limited to PET. Terms such as “strain”, “fungi” and “*Pseudozyma antarctica*” indicate continued interest in discovering diverse microbial sources of plastic-degrading enzymes. Compared with PET, the degradation of polyethylene, polypropylene, and polystyrene remains more difficult because these polymers lack readily hydrolyzable bonds and often require oxidation, surface activation, or long-term microbial colonization before substantial degradation can occur. For example, *Rhodococcus ruber* C208 was reported to colonize polyethylene films, form biofilms, and cause measurable polyethylene biodegradation, suggesting that biofilm formation is closely related to the degradation of resistant polyolefin plastics [63]. A subsequent study further showed that *R. ruber* preferentially maintained viable biofilms on polyethylene surfaces, supporting the importance of surface-associated growth during polyethylene biodegradation [64]. In marine conditions, the fungus *Zalerion maritimum* was shown to reduce the mass and size of polyethylene microplastic pellets under minimum nutrient conditions, with FTIR-ATR and NMR analyses indicating molecular-level changes [97]. However, these systems remain less mechanistically resolved than PETase/MHETase-mediated PET degradation. This contrast is scientifically important because it demonstrates that current application-oriented research is following two different routes: engineering defined hydrolases for controllable depolymerization of ester-containing plastics, and identifying oxidative microorganisms, biofilms, and consortia capable of initiating the transformation of resistant carbon–carbon-backbone plastics. Future application therefore requires polymer-specific strategies rather than a single universal biodegradation platform.

The recent bursts of “colonization” (strength = 6.53, 2017–2022) and “marine environment” (strength = 5.46, 2021–2023) are therefore particularly meaningful. They indicate that research is moving from isolated laboratory degradation tests toward the plastisphere perspective, in which plastic surfaces are treated as microbial habitats. The concept of the plastisphere was first clearly described by Zettler et al., who showed that marine plastic debris harbored distinct microbial communities compared with the surrounding seawater [108]. Subsequent studies further demonstrated that marine plastic surfaces can support biofilms composed of bacteria, fungi, archaea, algae, viruses, and protozoans, and that these communities are shaped by polymer type, environmental conditions, and exposure time [109,110]. However, recent marine plastisphere studies also caution that colonization does not necessarily mean degradation. For example, some plastisphere communities appear to be dominated by surface-attached generalists rather than microorganisms actively using plastic polymers as growth substrates [32]. This distinction is important for future research because many studies still infer biodegradation from surface changes, microbial growth, or community shifts without fully proving polymer depolymerization, monomer assimilation, or mineralization.

Previous bibliometric studies have examined plastic biodegradation at different levels of scope. Akinpelu and Nchu analyzed 290 Web of Science records published between 2000 and 2021, primarily mapping publication output, productive contributors, collaboration networks, and keyword structures [111]. Yang et al. examined 1500 publications on the biodegradation of petroleum-based synthetic plastics from 1991 to May 2023 and provided a longer-term overview of the development and major hotspots of the field [112]. Subsequent studies increasingly adopted more specific perspectives, including interactions between micro- or nanoplastics and microorganisms [113], microplastic biodegradation processes and mechanisms [114], potentially effective microorganisms for microplastic degradation [115], bacterial plastic degradation [116], and enzyme-oriented plastic biodegradation and process optimization [117]. These studies substantially clarified the growth and thematic diversification of plastic biodegradation research; however, their scopes generally emphasized either broad bibliometric mapping or a specific particle size, microbial group, degradation component, or technological route. In comparison, the present study analyzes 2959 manually screened Articles and Reviews published between 2000 and 2025 using a field-specific proximity search and integrates multiple bibliometric dimensions with a mechanistic mini-review. Its added value lies not merely in extending the observation period, but in linking publication growth, collaboration structures, citation patterns, and keyword evolution to a continuous biological framework comprising polymer aging, plastisphere formation, extracellular depolymerization or oxidative activation, uptake of soluble products, intracellular catabolic funneling, physiological regulation, ecological interactions, and bioremediation or upcycling. The present analysis also distinguishes hydrolyzable polyesters from resistant carbon–carbon-backbone plastics and considers bacteria, fungi, enzymes, and microbial consortia within the same interpretive framework. Therefore, this study moves beyond identifying publication patterns and emerging hotspots by explaining how the field is shifting from material-centered biodegradability testing toward polymer-specific mechanisms, environmentally realistic validation, and application-oriented resource recovery.

## 5. Conclusions and Future Perspectives

This study analyzed 2959 publications on microbial plastic biodegradation and related biodegradable polymer research published between 2000 and 2025. Annual publication output increased from 20 in 2000 to 617 in 2025, and 77.6% of the included publications were published between 2018 and 2025, demonstrating that the field has expanded particularly rapidly in recent years. China contributed the largest number of publications (892; approximately 30% of the dataset), whereas the United States showed the highest betweenness centrality (0.83), indicating that research productivity and international bridging influence represent distinct dimensions of scientific leadership. Citation and keyword analyses further showed that the knowledge base has shifted from general biodegradability and material-property studies toward polymer-specific enzymes, microbial mechanisms, environmental colonization, and application-oriented degradation.

These quantitative patterns provide a clear basis for future research priorities. The rapid increase in publications highlights the need for standardized criteria that distinguish surface deterioration from polymer depolymerization, microbial assimilation, and mineralization. The divergence between publication output and collaboration centrality indicates that stronger cross-regional cooperation is needed to support the sharing of strains, enzymes, protocols, and validation datasets. Meanwhile, the concentration of mechanistic advances around PET-degrading enzyme systems, together with the emergence of environmental and application-related keywords, indicates that future studies should expand polymer-specific research on more resistant plastics, validate degradation pathways under realistic environmental conditions, and evaluate their feasibility and ecological safety in bioremediation and upcycling applications.

Future research should first strengthen the discovery and validation of efficient plastic-degrading microorganisms and enzymes. The prominence of PET-degrading microorganisms, degrading enzymes, cutinase-like enzymes, fungi, and enzymatic degradation in highly cited publications and keyword evolution indicates that functional strains and enzyme systems remain central to this field [53,118]. PETase/MHETase-related systems have provided a relatively clear model for enzymatic plastic depolymerization, but degradation mechanisms for more resistant plastics such as polyethylene, polypropylene, and polystyrene remain less well defined. Therefore, future studies should identify new microorganisms and enzymes for representative plastics such as PET, polyethylene, polystyrene, polyurethane, and poly(lactic acid), and clarify their substrate specificity, catalytic efficiency, degradation products, and polymer-dependent limitations [119].

Second, mechanistic studies should move beyond surface deterioration and extracellular depolymerization. Complete microbial plastic biodegradation requires not only polymer cleavage, but also the formation of oligomers or monomers, microbial uptake, intracellular transformation, and, where possible, mineralization or conversion into biomass [20,120]. This is particularly important because microbial colonization, biofilm formation, FTIR changes, or surface erosion alone cannot fully prove true biodegradation. Future studies should combine chemical characterization, isotope tracing, enzyme assays, omics analysis, and metabolic pathway validation to distinguish plastic attachment from real plastic utilization and to identify the rate-limiting steps of biodegradation [120,121].

Third, microbial plastic biodegradation should be examined under more realistic environmental conditions. The increasing prominence of keywords such as “microplastics,” “pollution,” “colonization,” “marine environment,” and “behavior” suggests that current research frontiers are closely associated with environmental fate, biofilm formation, and ecological risks [122,123]. Plastics in natural environments are affected by aging, fragmentation, UV exposure, additives, coexisting pollutants, nutrient limitation, and complex microbial communities. Future work should therefore incorporate soil, freshwater, marine, composting, wastewater-treatment, and landfill-related systems to determine whether degradation mechanisms observed under controlled laboratory conditions can also operate in complex environments [66,67].

Fourth, biodegradable material design and application-oriented validation require more systematic evaluation. Keywords such as “biodegradable,” “composites,” “poly(lactic acid),” “chitosan,” and “nanoparticles” indicate that microbial plastic biodegradation research is increasingly connected with material innovation and circular resource use [124,125]. Future studies should not only assess whether a material is degradable, but also evaluate degradation rate, environmental stability, transformation products, ecological safety, process feasibility, and practical applicability [124]. In particular, microbial consortia, engineered enzymes, bioremediation strategies, composting systems, and plastic upcycling approaches should be validated under realistic and environmentally safe application scenarios [121].

Overall, microbial plastic biodegradation research has gradually developed from early studies of polymer degradation behavior and material properties into a broader interdisciplinary field integrating microbial mechanisms, enzymatic catalysis, environmental fate, pollution control, and sustainable material applications. Future progress will depend on closer integration among mechanistic microbiology, polymer material design, environmental risk assessment, and application-oriented validation. Such integration will help move the field from descriptive degradation observations toward a more complete framework for plastic pollution control and circular resource utilization.

## Figures and Tables

**Table 1:** Top 10 countries/regions by publication output in microbial plastic biodegradation research.

Rank	Top 10 Countries/Regions				Top 10 Institutions			Top 10 Authors		
	Country	Publications	Betweenness Centrality	Proportion of Total Publications	Institution (Country)	Publications	Citations	Author (Country)	Publications	Citations
1	China	892	0.15	0.30	Chinese Academy of Sciences (China)	144	6770	Weiliang Dong (China)	36	838
2	India	369	0.09	0.12	University of Chinese Academy of Sciences (China)	56	2250	Wei-Min Wu (United States)	33	4234
3	USA	301	0.83	0.10	National Institute of Advanced Industrial Science and Technology (Japan)	43	3314	Ren Wei (Germany)	32	1829
4	Japan	238	0.04	0.08	Nanjing Tech University (China)	41	962	Jie Zhou (China)	24	1551
5	Germany	174	0.26	0.06	Stanford University (United States)	38	4496	Yuka Sameshima-Yamashita (Japan)	22	675
6	South Korea	167	0.09	0.06	Harbin Institute of Technology (China)	35	2100	Hiroko Kitamoto (Japan)	21	318
7	Italy	118	0.55	0.04	Northwest Agriculture & Forestry University (China)	34	1955	Takashi Watanabe (Japan)	19	630
8	Spain	107	0.04	0.04	Tongji University (China)	32	2497	Ken-ichi Kasuya (Japan)	18	699
9	Canada	91	0.16	0.03	Shandong University (China)	30	643	Motoo Koitabashi (Japan)	17	630
10	The United Kingdom	88	0.24	0.03	Tianjin University (China)	26	1215	Min Jiang (China)	17	544

**Table 2:** Top 15 journals by publication output in microbial plastic biodegradation research.

Journal	Publications	Country	IF (2025)	JCR (2025)
*Journal of Hazardous Materials*	193	Netherlands	11.3	Q1
*Science of the Total Environment*	129	Netherlands	8.0	Q1
*Polymer Degradation and Stability*	92	United Kingdom	7.4	Q1
*Polymers*	59	Switzerland	4.9	Q1
*Frontiers in Microbiology*	57	Switzerland	4.5	Q1
*Bioresource Technology*	51	Netherlands	9.0	Q1
*Environmental Pollution*	48	United Kingdom	7.3	Q1
*International Biodeterioration & Biodegradation*	47	United Kingdom	4.1	Q2
*Journal of Polymers and the Environment*	41	United States	5.0	Q1
*Environmental Science & Technology*	40	United States	11.3	Q1
*Journal of Applied Polymer Science*	36	United States	2.8	Q3
*Applied Microbiology and Biotechnology*	35	Germany	4.3	Q1
*International Journal of Biological Macromolecules*	35	Netherlands	8.5	Q1
*Journal of Environmental Management*	32	United Kingdom	8.4	Q1
*Biodegradation*	31	Netherlands	3.2	Q2

**Table 3:** Top 10 most-cited publications representing the knowledge base of microbial plastic biodegradation research.

Title	Year	Journal	First Author	Total Citations	Reference
Biological degradation of plastics: A comprehensive review	2008	*Biotechnology Advances*	Shah, Aamer Ali	1907	[81]
An engineered PET depolymerase to break down and recycle plastic bottles	2020	*Nature*	Tournier, V	1665	[61]
Biodegradability of Plastics	2009	*International Journal of Molecular Sciences*	Tokiwa, Yutaka	1207	[82]
Polyethylene and biodegradable mulches for agricultural applications: a review	2012	*Agronomy for Sustainable Development*	Kasirajan, Subrahmaniyan	1064	[83]
Biodegradation of poly (vinyl alcohol) based materials	2003	*Progress in Polymer Science*	Chiellini, E	857	[84]
Biodegradation of bioplastics in natural environments	2017	*Waste Management*	Emadian, S. Mehdi	810	[85]
Microbial and Enzymatic Degradation of Synthetic Plastics	2020	*Frontiers in Microbiology*	Mohanan, Nisha	752	[86]
Evidence of Polyethylene Biodegradation by Bacterial Strains from the Guts of Plastic-Eating Waxworms	2014	*Environmental Science & Technology*	Yang, Jun	739	[87]
Review of recent advances in the biodegradability of polyhydroxyalkanoate (PHA) bioplastics and their composites	2020	*Green Chemistry*	Meereboer, Kjeld W.	703	[88]
Bacterial polyesters: Biosynthesis, biodegradable plastics and biotechnology	2005	*Biomacromolecules*	Lenz, RW	650	[89]

## Data Availability

No new data were created or analyzed in this study. Data sharing is not applicable to this article.

## Supplementary Materials

The following supporting information can be downloaded at: https://www.mdpi.com/article/10.3390/microorganisms14081695/s1, Supplementary File S1: Full records and cited references exported from Web of Science.
