# Supplementary material for: Global Hotspots and Trends in Microbial Plastic Biodegradation for Plastic Waste Management: A Mini-Review and Bibliometric Analysis
Source: Microorganisms. 2026 Aug 2;14(8):1695. doi: 10.3390/microorganisms14081695 (PMC13515210; doi:10.3390/microorganisms14081695)
Supplement: Supplementary file 1 [file microorganisms-14-01695-s001.zip › microorganisms-4455518-supplementary.pdf]

Supplementary File S1: Full records and cited references exported from Web of Science.

```
(
 TI=(
  (
   plastic OR plastics OR microplastic* OR nanoplastic* OR
   "plastic waste" OR "plastic wastes" OR
   polyethylene OR "polyethylene terephthalate" OR
   "poly(ethylene terephthalate)" OR polypropylene OR polystyrene OR
   polyurethane OR "polyvinyl chloride" OR "poly(vinyl chloride)" OR
   "polyvinyl alcohol" OR "poly(vinyl alcohol)" OR nylon OR polyamide*
  )
  NEAR/8
  (
   biodegrad* OR biodeteriorat* OR biofragment* OR bioassimilat* OR
   "biological degradation" OR "microbial degradation" OR
   "enzymatic degradation" OR depolymer* OR mineraliz* OR mineralis*
  )
 )
 OR
 AB=(
  (
   plastic OR plastics OR microplastic* OR nanoplastic* OR
   "plastic waste" OR "plastic wastes" OR
   polyethylene OR "polyethylene terephthalate" OR
   "poly(ethylene terephthalate)" OR polypropylene OR polystyrene OR
   polyurethane OR "polyvinyl chloride" OR "poly(vinyl chloride)" OR
   "polyvinyl alcohol" OR "poly(vinyl alcohol)" OR nylon OR polyamide*
  )
  NEAR/8
  (
   biodegrad* OR biodeteriorat* OR biofragment* OR bioassimilat* OR
   "biological degradation" OR "microbial degradation" OR
   "enzymatic degradation" OR depolymer* OR mineraliz* OR mineralis*
  )
 )
 OR
 AK=(
  (
   plastic OR plastics OR microplastic* OR nanoplastic* OR
   "plastic waste" OR "plastic wastes" OR
   polyethylene OR "polyethylene terephthalate" OR
   "poly(ethylene terephthalate)" OR polypropylene OR polystyrene OR
   polyurethane OR "polyvinyl chloride" OR "poly(vinyl chloride)" OR
```

```
    "polyvinyl alcohol" OR "poly(vinyl alcohol)" OR nylon OR polyamide*
   )
   NEAR/8
   (
    biodegrad* OR biodeteriorat* OR biofragment* OR bioassimilat* OR
    "biological degradation" OR "microbial degradation" OR
    "enzymatic degradation" OR depolymer* OR mineraliz* OR mineralis*
   )
  )
)
AND
(
 TI=(
   (
    microb* OR bacteri* OR fungal OR fungi OR fungus OR yeast* OR
    actinomycet* OR biofilm* OR "microbial consortium" OR
    "microbial consortia" OR "mixed culture" OR "mixed cultures" OR
    "gut microbiota" OR "gut microbiome" OR "intestinal microbiota" OR
    enzym* OR enzyme* OR hydrolase* OR cutinase* OR esterase* OR lipase* OR
    laccase* OR oxidase* OR oxygenase* OR depolymerase* OR PETase OR MHETase
   )
   NEAR/10
   (
    biodegrad* OR degrad* OR depolymer* OR hydroly* OR mineraliz* OR
    mineralis* OR assimilat*
   )
 )
 OR
 AB=(
   (
    microb* OR bacteri* OR fungal OR fungi OR fungus OR yeast* OR
    actinomycet* OR biofilm* OR "microbial consortium" OR
    "microbial consortia" OR "mixed culture" OR "mixed cultures" OR
    "gut microbiota" OR "gut microbiome" OR "intestinal microbiota" OR
    enzym* OR enzyme* OR hydrolase* OR cutinase* OR esterase* OR lipase* OR
    laccase* OR oxidase* OR oxygenase* OR depolymerase* OR PETase OR MHETase
   )
   NEAR/10
   (
    biodegrad* OR degrad* OR depolymer* OR hydroly* OR mineraliz* OR
    mineralis* OR assimilat*
   )
 )
 OR
```

```
AK=(
  (
    microb* OR bacteri* OR fungal OR fungi OR fungus OR yeast* OR
    actinomycet* OR biofilm* OR "microbial consortium" OR
    "microbial consortia" OR "mixed culture" OR "mixed cultures" OR
    "gut microbiota" OR "gut microbiome" OR "intestinal microbiota" OR
    enzym* OR enzyme* OR hydrolase* OR cutinase* OR esterase* OR lipase* OR
    laccase* OR oxidase* OR oxygenase* OR depolymerase* OR PETase OR MHETase
  )
  NEAR/10
  (
    biodegrad* OR degrad* OR depolymer* OR hydroly* OR mineraliz* OR
    mineralis* OR assimilat*
  )
 )
)
```
